# Electron Injection in Metal Assisted Chemical Etching as a Fundamental Mechanism for Electroless Electricity Generation

**DOI:** 10.1021/acs.jpclett.2c01302

**Published:** 2022-06-16

**Authors:** Shengyang Li, Kexun Chen, Ville Vähänissi, Ivan Radevici, Hele Savin, Jani Oksanen

**Affiliations:** † Engineered Nanosystems Group, School of Science, Aalto University, Tietotie 1, Espoo, 02150, Finland; ‡ Department of Electronics and Nanoengineering, Aalto University, Tietotie 3, Espoo, 02150, Finland

## Abstract

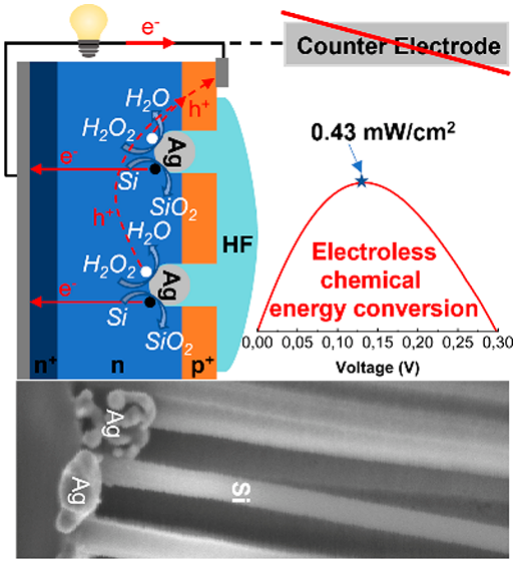

Metal-assisted chemical etching (MACE) is a widely applied process for fabricating Si nanostructures. As an electroless process, it does not require a counter electrode, and it is usually considered that only holes in the Si valence band contribute to the process. In this work, a charge carrier collecting p–n junction structure coated with silver nanoparticles is used to demonstrate that also electrons in the conduction band play a fundamental role in MACE, and enable an electroless chemical energy conversion process that was not previously reported. The studied structures generate electricity at a power density of 0.43 mW/cm^2^ during MACE. This necessitates reformulating the microscopic electrochemical description of the Si-metal-oxidant nanosystems to separately account for electron and hole injections into the conduction and valence band of Si. Our work provides new insight into the fundamentals of MACE and demonstrates a radically new route to chemical energy conversion by solar cell-inspired devices.

Metal assisted chemical etching (MACE, also known as metal catalyzed electroless etching, MCEE) is widely employed to fabricate silicon nanostructures (e.g., porous Si, black Si, Si nanowires) and has been studied for over 20 years.^1−13^ Currently two main routes for MACE are being studied. One is the single-step MACE which uses metal salts as the oxidants and thus combines the metal deposition and etching process into one step,^2,14^ while the other is a two-step process that separates the metal deposition process from the etching process.^15^ So far, various metals (e.g., Ag, Au, Pt) with different geometries (e.g., nanoparticles, thin film) have been successfully used as the catalyst in MACE.^16,17^ The MACE process is based on the highly site-selective oxidation of Si when it is brought into contact with metal nanoparticles and oxidants (typically hydrogen peroxide, H_2_O_2_) in an HF solution, and it can produce high-aspect-ratio silicon nanostructures (SiNSs). Due to the simplicity, versatility, and scalability of the MACE process, it has been widely used to fabricate SiNSs, especially for advanced energy conversion and storage, biomedicine, and sensor applications.^18−20^ The MACE process of Si has been readily explained by the formation of microscopic wireless and short circuited galvanic cells in the Si–metal–oxidant nanosystems as illustrated in Figure 1.^21−24^ The overall reaction is considered to proceed through two main steps: (1) the reduction of H_2_O_2_ molecules into water injecting holes transferring via silver nanoparticles (AgNPs) into the valence band of Si and (2) the oxidation of Si into SiO_2_ by the injected holes.^25−27^ The formed SiO_2_ is readily removed by the HF etchant so that the electrochemical reactions on the Si surface can continue, and SiNSs are formed in the end.

**Figure 1:**
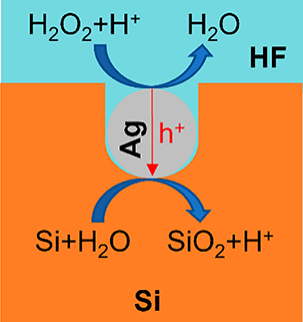
Established model of the microscopic galvanic cell formed during the fabrication of SiNS by the MACE process.

In addition to the reactions involving the valence band, as previously reported for MACE, also the chemical injection of electrons into the conduction band of semiconductors is well-known in the phenomena of electrogenerated chemiluminescence and photocurrent doubling present in a wide range of semiconductor materials.^28−32^ While these studies require the use of a counter electrode to host the corresponding electrochemical reduction reactions, charge transfer processes occurring simultaneously between both semiconductor bands and suitable redox couples in electrolytes without counter electrodes are also widely reported. Such bipolar redox processes form the basis of, for example, photocatalytic water splitting by particulate photocatalysts and chemiluminescence of semiconductor quantum dots (QDs).^33−36^ From earlier works, however, it is not evident if electron transfer processes involving the conduction band also contribute to MACE, and when more closely considering the related redox reactions, an obvious question arises: Are similar charge carrier injection processes between both bands of Si and the redox couples present also in the Si–metal–oxidant nanosystems of MACE? And if they are, how can they be observed and possibly even utilized?

In this work, we designed a p–n junction Si cell coated with AgNPs to probe the charge carrier injection processes during the MACE process. Internally, this resembles the functionality of a p–n junction Si solar cell where light generates photoinduced electron–hole pairs that are separated by the p–n junction generating electricity. Current density–voltage (*J*–*V*) and chronoamperometry measurements were carried out on the cell during MACE. The measurements show that upon exposure to MACE the cells can generate electricity at a power density of ∼0.43 mW/cm^2^. Based on the experimental results, a formulation separately accounting for electron and hole injections into the conduction and valence band of Si with the assistance of silver nanoparticles is proposed for describing the microscopic electrochemical characteristics of the Si–metal–oxidant nanosystems formed during the MACE process. In the end, the hybrid functionality of the cell is also demonstrated by showing that the cell could harvest both optical and chemical energy. Our work provides valuable insight on the microscopic charge transfer processes taking place during MACE. More interestingly, however, this also demonstrates a radically new route to chemical energy conversion by a semiconductor p–n junction device using an electroless process that does not involve a counter electrode.

For the experiments, Si p–n junction samples were fabricated. The fabrication followed a simplified version of the previously reported process flow used for fabricating Si solar cells (see the details in Supporting Information).^37^ The samples were encapsulated with hot glue, leaving an active area of 1–1.3 cm^2^ exposed on the front side surface. After this the samples were divided into two batches. The first batch continued directly to the measurements but for the second batch, AgNPs were deposited on the active area by the method reported before: the encapsulated samples were immersed in the solution of 5 mM silver nitrate and 6 M HF for 8 min.^24^ During this time, the displacement reaction of Si with silver ions took place and AgNPs with a diameter of 50–200 nm were deposited on the Si surface. The surface SEM image of the sample after AgNPs deposition is shown in Figure 2a. From hereafter, the samples with and without AgNPs are denoted as AgNPs-p-n-Si and Planar-p-n-Si, respectively.

**Figure 2:**
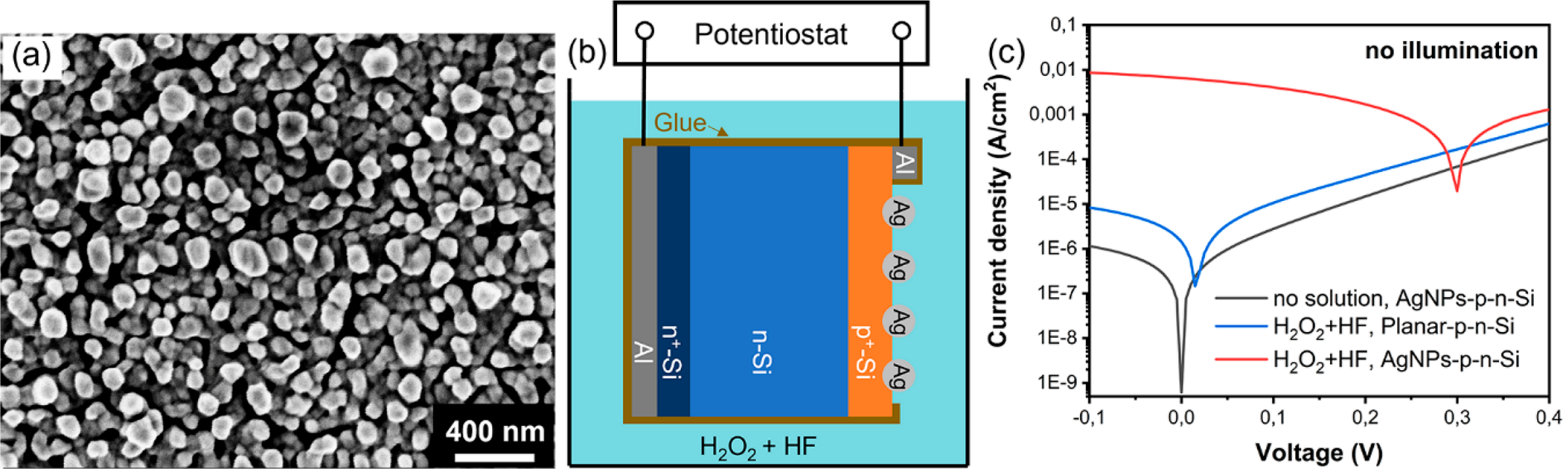
(a) Surface SEM image of AgNPs-p-n-Si. (b) Schematic illustration of the set up used for electrochemical measurements. (c) *J*–*V* curves of the samples in different conditions.

The setup used for electrochemical measurements is schematically shown in Figure 2b. The front and back side Al contacts of the sample were connected to a potentiostat by insulated copper wires passing through the encapsulation. The *J*–*V* measurements were performed in the dark with the sample placed in a beaker under controlled exposure to chemicals allowing creation of a MACE environment for the samples and measurement of the *J*–*V* curves of the samples during MACE. The results are shown in Figure 2c. Before exposure to the solution both AgNPs-p*-*n-Si and Planar-p-n-Si show a standard rectifying curve with an open circuit voltage (*V*_oc_) of zero. Quite remarkably, when the samples are exposed to an aqueous solution of 0.4 M H_2_O_2_ and 6 M HF as typically used for the MACE of Si,^38^ the *J*–*V* characteristics change dramatically, and electricity is generated. The AgNPs-p-n-Si exhibits a *V*_oc_ of 0.3 V and a short circuit current density (*J*_sc_) of 6.5 mA/cm^2^. Also, the Planar-p-n-Si exhibits a small but clearly measurable *V*_oc_ of 15 mV and *J*_sc_ of 1.4 μA/cm^2^. Because the measurements were performed in complete darkness, we attribute the generation of electricity by the p–n junction Si samples to the excitation of Si by the chemical energy released during the chemical etching.

In the MACE system, the main two redox couples are Si/SiO_2_ with a standard electrode potential *E*^0^ = −0.91 V vs SHE (standard hydrogen electrode) and H_2_O_2_/H_2_O with a standard electrode potential *E*^0^ = 1.77 V vs SHE,^39,40^ as shown in Figure 3a. Considering the band edge positions of Si, both electron and hole injections from the redox couples into the conduction and valence bands are then possible. We propose the following mechanism for the generation of excitation in Si, leading to the generation of electricity when the sample is in contact with the solution of H_2_O_2_ and HF. Due to the low oxidation potential of Si, the surface Si–Si bonds interact with water molecules and loose electrons by injecting free electrons into the conduction band (CB). This reaction forms SiO_2_ on the surface and releases protons to the solution in the process as shown by the *anodic half reaction*
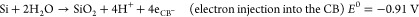
Concurrently, the H_2_O_2_ molecule in the solution as a strong oxidant gains electrons from the valence band (VB) of Si (i.e., injects holes into the VB) due to its high reduction potential. As a result, the molecules reduce into water in the process as shown by the *cathodic half reaction*
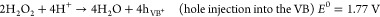
The formed SiO_2_ is readily removed by the HF etchant in the solution, exposing fresh Si surface. That keeps the two electrochemical reactions on the Si surface going thus allowing continued electron and hole injections to the conduction and valence bands (i.e., excitation of Si) with the overall reaction
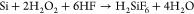


**Figure 3:**
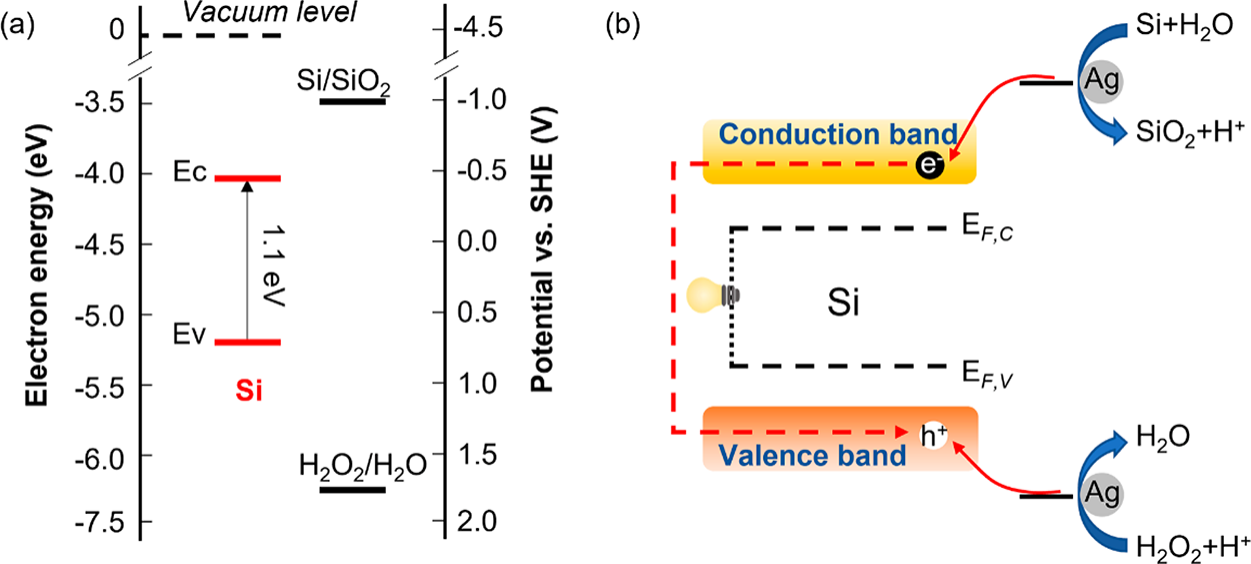
(a) Energy level diagram of Si in contact with an aqueous solution containing H_2_O_2_ at pH 0. The divalent oxidation of Si during the MACE, that is Si + 6F^–^ + 2H^+^ →SiF_6_^2–^ + H_2_ + 2e^–^ with a standard electrode potential of *E*^0^ = −1.2 V vs SHE, was also proposed by Chartier et al.^41^ For simplicity, we only consider the tetravalent oxidation of Si here, but the main principle is the same. (b) The excitation of Si by chemical energy released during MACE allows the generation of electricity in an electroless process that does not involve a counter electrode.

As shown in Figure 2c, AgNPs-p-n-Si exhibits a much higher *V*_oc_ and *J*_sc_ compared with Planar-p-n-Si. Considering the proposed mechanism, the difference can be attributed to the catalytic effect of AgNPs to both electrochemical reactions shown above. Nobel metal nanoparticles have been widely used as highly effective catalysts for various electrochemical reactions in fuel cells and (photo)electrocatalysis.^42,43^ Here, the AgNPs catalyze the H_2_O_2_ reduction and Si oxidation electrochemical reactions and significantly improve the kinetics of these electrochemical reactions, that is, the carrier injection rate, causing the current density to increase by 3 orders of magnitude.

Figure 3b schematically illustrates how the injection of electrons and holes from the redox couples Si/SiO_2_ and H_2_O_2_/H_2_O into the conduction and valence band of Si proceeds with the assistance of AgNPs during MACE. The injection of excess charge carriers into Si results in a significant nonequilibrium concentration of minority carriers, giving rise to the splitting of the Fermi level into conduction and valence band quasi-Fermi levels E_F,C_ and E_F,V_. These chemical-energy-induced electron–hole pairs are then separated and transported to the external circuit by diffusion outside the junction area and drift in the junction area, causing the generation of electricity in an electroless process.

Chronoamperometry measurements were carried out on AgNPs-p-n-Si at short-circuit conditions in the aqueous HF solution with and without H_2_O_2_, and the results are shown in Figure 4a. While without H_2_O_2_ as oxidant there was no obvious electric current generation, a continuous electric current was generated with the solution containing H_2_O_2_. The short-circuit current density of the sample drops abruptly from 6.8 mA/cm^2^ to around 2 mA/cm^2^ during the first 5 min and then remains around 1 mA/cm^2^ after 15 min of etching. The reduction of the current density can originate from the slowing down of the electrochemical reactions due to the mass-transfer limitations, from a decrease in the collection efficiency of the induced carriers due to the increase of the distance that the induced carriers need to diffuse to reach the junction, the increase in resistivity of the p-doped layer due to pore formation in the 1.5 μm thick p-doped layer during the etching,^44^ and the increase of the surface area enabling surface defects. Both processes may change relatively quickly when the etching proceeds through the p-doped top layer during the first couple of minutes of the etching. After etching through the top p-layer, both processes are expected to change more slowly, resulting in a relatively slow decrease in the current. Due to the high catalytic activity of Ag, it is additionally noted that Si beneath the AgNPs is preferentially used as a fuel to generate electricity, leading to the formation of Si nanostructuration reaching a total depth 25 μm (corresponding to an etch rate of 0.83 μm/min) after the etching, as illustrated in Figure 4b,c showing the cross-sectional SEM images of AgNPs-p-n-Si after the chronoamperometry measurement.

**Figure 4:**
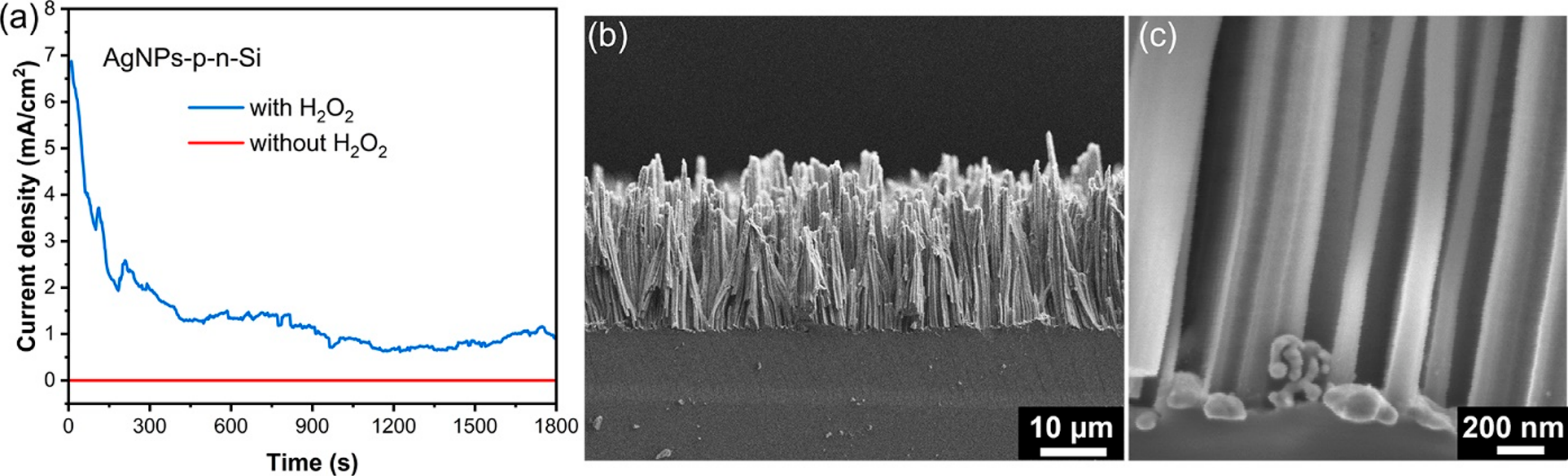
(a) Chronoamperometry of AgNPs-p-n-Si at short-circuit conditions in the aqueous HF solution with and without H_2_O_2_. (b,c) cross sectional SEM image of AgNPs-p-n-Si after the chronoamperometry measurement.

Based on the above experimental results and analysis, MACE proceeds through a bipolar reaction mechanism involving both energy bands of Si. For unconnected structures or structures without an inner junction as used in most previous studies, the injected electrons and holes during MACE would just recombine without any externally observable changes in the MACE process, or the possibility to observe the excitation.

To show that the device can harvest both optical and chemical energy, we also measured the *J*–*V* curve of AgNPs-p-n-Si when exposed to 0.1 mW/cm^2^ white light illumination before exposing it to the MACE solution. The *J*–*V* curves for the two cases are shown in Figure 5a. At this optical excitation level, the *V*_oc_ of the cell is 0.28 V and *J*_sc_ is 52 μA/cm^2^. Figure 5b shows the power density–voltage curves in the two cases. Under chemical excitation, the maximum power density of the cell is 0.43 mW/cm^2^ at a voltage of 0.135 V, and the maximum power density of the cell under the optical excitation is 0.008 mW/cm^2^ at a voltage of 0.2 V. On the basis of the experimental results and making the assumptions that the etching rate is constant, and that half of surface area is etched on average during MACE, the energy generation of per mole Si and the average carrier collection efficiency of the device are estimated to be 7.5 × 10^3^J/mol and 6.4%, respectively (see the calculation details in the Supporting Information). Figure 5 panels c and d schematically illustrate the two setups. When the sample is illuminated, the electron–hole pairs are generated deep within the sample (Figure 5c). During the chemical excitation both types of carriers are selectively generated at the Si surface in contact with the AgNPs even in the absence of light (Figure 5d). In both cases, however, the electricity generation mechanism is essentially based on the charge separation process of the p–n junction. To study the influence of the deposited AgNPs on the light absorption, we also compared the *J*–*V* curves of both Planar-p-n-Si and AgNPs-p-n-Si samples under simulated sun light (100 mW/cm^2^) illumination. The results are shown in Figure S1. While both samples exhibit approximately the same *V*_oc_ of 0.52 V, the Planar-p-n-Si exhibits a *J*_sc_ of 31.1 mA/cm^2^ which is 1.5 times as large as the 21.4 mA/cm^2^ produced by the AgNPs-p-n-Si sample. The lower *J*_sc_ of AgNPs-p-n-Si indicates that the silver nanoparticles reduce the light intensity in the junction by around 30% due to the blocking of the light by the deposited AgNPs layer.

**Figure 5:**
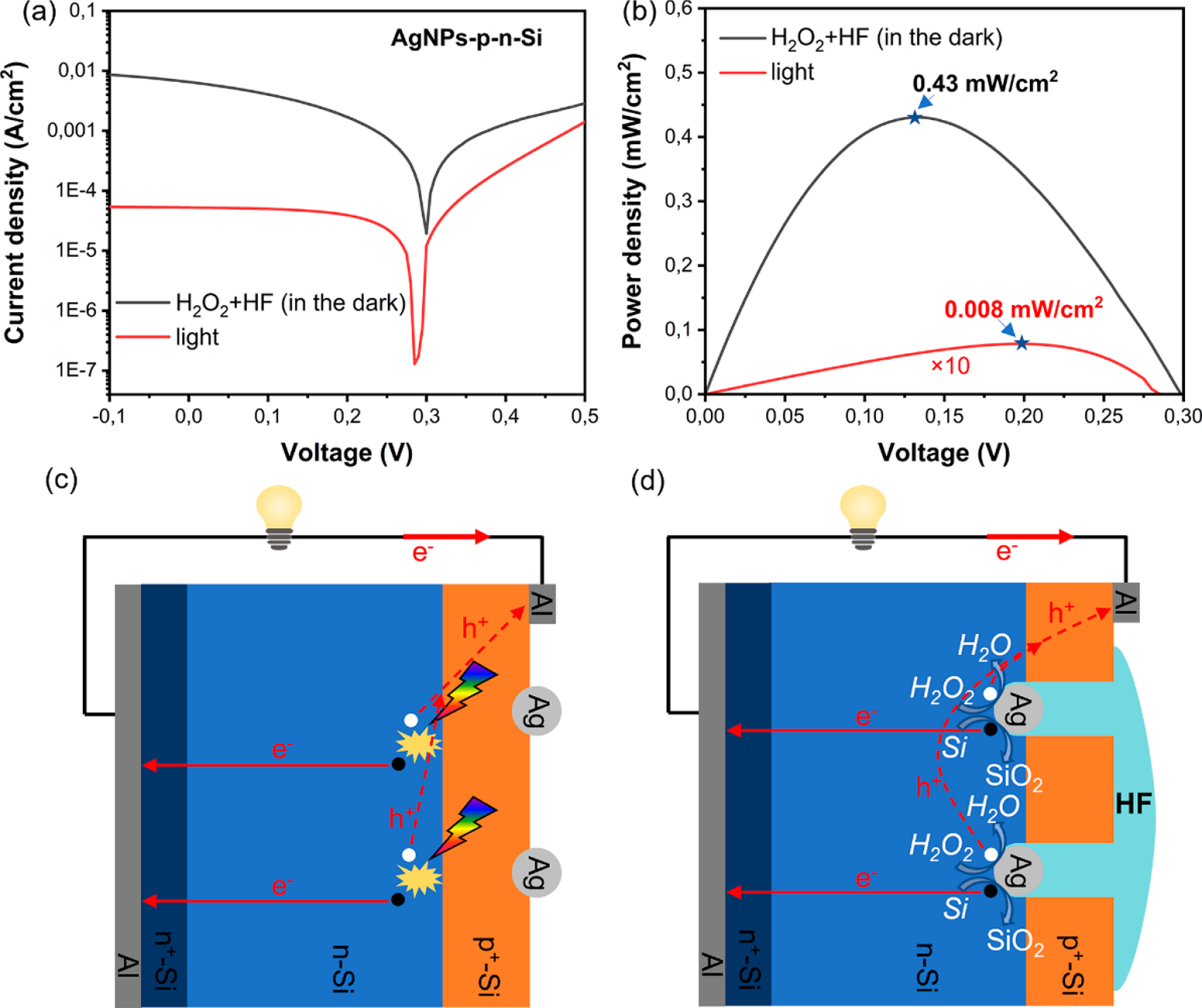
(a) *J*–*V* and (b) power density–voltage curves of the AgNPs-p-n-Si under 0.1 mW/cm^2^ white light illumination and under exposure to a H_2_O_2_ and HF solution in the dark. (c) Schematic illustration of the electricity generation mechanism by AgNPs-p-n-Si under illumination and (d) in contact with the H_2_O_2_ and HF solution.

In summary, we have demonstrated the participation of both conduction and valence bands in the MACE process as well as the extraction of chemical energy released during MACE of Si by a p–n junction Si cell coated with AgNPs in an electroless process that does not involve a counter electrode. During the MACE, the cell can produce energy at a power density of ∼0.43 mW/cm^2^. This shows that MACE of Si fundamentally relies on charge carrier injection to both bands of Si from the respective redox couples in the Si–metal–oxidant nanosystems, leading to the chemical excitation (generation of electron–hole pairs) of Si. The AgNPs deposited on the Si surface as highly active catalysts significantly increase the carrier injection rates and thus significantly improve the performance of the cell. Revealing the detailed dynamics of the bipolar charge transfer process provides substantial added insight for the microscopic electrochemistry of AgNPs assisted chemical etching of Si. While the p–n junction structure is usually used to convert optical energy into electricity, our work demonstrates that it can also be used to harvest chemical energy. This can open the door to direct conversion of chemical energy using solar cell inspired devices.

## Supporting Information Available

Additional experimental details for preparation of the p–n junction Si samples, silver nanoparticles (AgNPs) deposition, and device performance characterization; details of estimating the molar energy generation and carrier collection efficiency of the device; *J*–*V* curves of samples under 100 mW/cm^2^ illumination (PDF)
