# Supplementary material for: Electron Injection in Metal Assisted Chemical Etching as a Fundamental Mechanism for Electroless Electricity Generation
Source: J Phys Chem Lett. 2022 Jun 16;13(24):5648–53. doi: 10.1021/acs.jpclett.2c01302 (PMC9234978; doi:10.1021/acs.jpclett.2c01302)
Supplement: Supplementary file 1 — jz2c01302_si_001.pdf [file jz2c01302_si_001.pdf]

Supporting Information

Electron Injection in Metal Assisted Chemical Etching as a Fundamental Mechanism for Electroless Electricity Generation

Shengyang Li,[1]* *Kexun Chen,*[2] *Ville Vähänissi,*[2] *Ivan Radevici,*[1] *Hele Savin,*[2] *Jani Oksanen*[1]

[1]Engineered Nanosystems Group, School of Science, Aalto University, Tietotie 1, Espoo, 02150, Finland

[2]Department of Electronics and Nanoengineering, Aalto University, Tietotie 3, Espoo, 02150, Finland

Email: shengyang.li@aalto.fi

Experimental Details

Preparation of the p-n junction Si samples

Double side polished phosphorous-doped n-type Si (111) substrate with resistivity of 3.0 ± 2.0 Ω cm and thickness of 280 ± 15 um were used to prepare the p/n junction Si sample. The wafers were first cleaned by standard RCA-1 and RCA-2 cleaning. Then, front side emitter layer was formed by boron ion implantation with the implantation energy of 10 KeV, tilt angle of 7° and dose of 3×10^{15} cm^{-2}. In order to obtain a good ohmic contact, a back-side n^+ layer was formed by phosphorous ion implantation with the implantation energy of 20 KeV, tilt angle of 7° and dose of 4.75×10^{15} cm^{-2}. Dopant activation, both for the emitter p^+ layer and the contact n^+ layer, was achieved via annealing at 1050 °C for 20 min under flowing nitrogen, followed by a 20 min dry oxidation at the same temperature, which resulted in front side emitter sheet resistance of 45 Ω/□ and the formation of a layer of SiO_2 with thickness of 15 nm on both sides of the wafer. A lithography and BHF etching were carried out for the front and back side to create contact openings in the SiO_2 layer protecting the other areas of the sample. This was followed by the deposition of Al films with a thickness of 0.3 um and 1 um on the front and backside of the wafers by magnetron sputtering (MRC-903), respectively. After that another lithography and an Al etch were applied to pattern the Al on the front surface. Thereafter, a final lithography

process and BHF etching were applied to the wafers to remove the remaining SiO_2 on the front side of the wafers. Finally, the wafers were annealed in forming gas at 425 °C for 20 min to sinter the Al contacts.

Silver nanoparticles (AgNPs) deposition

The wafers were cut to small pieces (1.6×2.1 cm^2) for AgNPs deposition and the later performance characterizations. The samples were encapsulated by hot glue before AgNPs deposition. Specifically, two insulated copper wires were attached to the front and backside Al contacts of the sample using conductive silver paste. The two copper wires were passed through two glass tubes covered with hot glue, and the samples were insulated with hot glue apart from the 1-1.3 cm^2 active area of the sample that was left exposed. Then, the sample was immersed in the aqueous solution of 5 mM $AgNO_3$ and 6 M HF for 8 minutes, during which the AgNPs were deposited on the active area of the sample by the displacement reaction of Si with Ag ions. After that the sample was thoroughly rinsed by DI water and dried by N_2.

Device performance characterization

The front and back side Al contacts of the sample were connected by two copper wires to a two-channel source-meter (Keithley 2612B) used as a potentiostat. The samples were put in a beaker with 0.4 M H_2O_2 and 6 M HF solution. The J-V measurements were performed in the dark by scanning from -1 to 1 V with a scan rate of 50 mV/s. Chronoamperometry measurements were performed at short-circuit condition under darkness in the same solution with J-V measurements for 30 minutes. For the J-V measurements under illumination, the samples were illuminated by a fluorescent lamp of a fume hood. The light intensity on the sample surface was measured by a Si photodiode power sensor (S130VC, Thermolab) and was found to be 0.1 mW/cm^2. The samples were also illuminated by a solar simulator (HAL-320 W, Asahi Spectra), and the light intensity on the sample was calibrated to 100 mW cm^{-2} by using the Si photodiode power sensor.

Calculation of the molar energy content (E_M) and the carrier collection efficiency (Φ_{col}) of the device

The SEM image (Figure 4b in manuscript) shows that Si nanostructures with a thickness of 25 µm were formed on the sample surface after 30 minutes etching. This corresponds to an etching rate of 0.83 µm/min, i.e., k=13.8 nm/s. We assume that the etching rate is constant, and that

half of surface area is etched on average during MACE. The lattice constant of Si is a=5.43 Å, and there are N=8 atoms per unit cell. Molar etching rate of Si (M) per unit area of the device is then given by

$M = 1/2 \times k \times N/(a^3 \times N_A)$,

where $N_A = 6.02\times10^{23}$ mol^{-1} is the Avogadro constant. The calculated value of M is 5.73×10^{-8} moles/cm^{2}s. The measured peak power density of the device is 0.43 mW/cm^2, i.e. 4.3×10^{-4} J/cm^{2}s. This leads to the energy generation per mole Si (E_M) of 7.5×10^3J/mol. Obviously, the power density of the device also decreases as the etching proceeds and the current decreases (shown in Figure 4a); here we only calculated the power generation of per mole Si of the device in the beginning of the etching, where we measured the peak power density.

Correspondingly for quantum efficiency, the number of etched Si atoms per unit area (N_{Si}) averaged over the 30-minute etching is

$N_{Si} = 1/2 \times d \times N/a^3$

where d = 25 µm is the etching depth. If we assume that one Si releases 4 electrons, then the number of electrons released during the 30-minute etching is $N_r = 4\times N_{Si}$, i.e. N_r is 2.5×10^{20}/cm^2.

From the chronoamperometry curves (Figure 4a in manuscript), we estimate the number of electrons collected from the unit area of the device during the 30-minute etching (N_c) by integrating the curve, getting 1.6 $\times10^{19}$/cm^2.

From these numbers, the average carrier collection efficiency of the device would be $\Phi_{col} = N_c/N_r$ = 6.4%. Because the collected current decreases with etching (as shown in Figure 4a in manuscript), the charge carrier collection efficiency in the beginning of the etching is likely to be significantly higher than this average value.

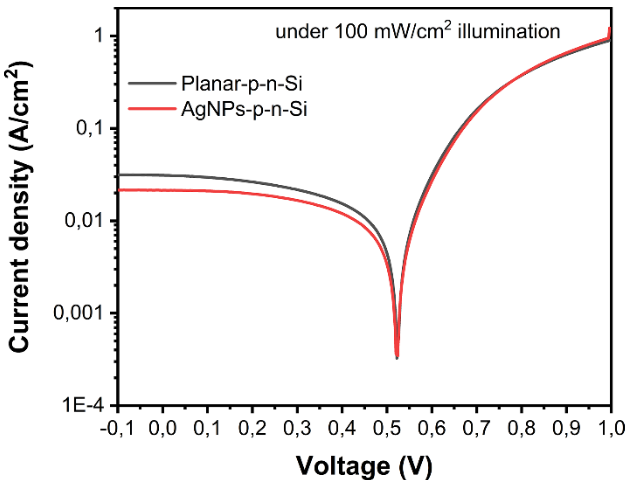

Figure S1. J-V curves of the Planar-p-n-Si and AgNPs-p-n-Si under 100 mW/cm^2 simulated sun light illumination.
